# Impaired detrusor contractility due to venlafaxine use

**DOI:** 10.4103/0970-1591.44276

**Published:** 2008

**Authors:** J. Torgovnick, N. K. Sethi, P. K. Sethi, E. Arsura

**Affiliations:** 1 Department of Neurology, Saint Vincent's Hospital and Medical Centers, New York, NY U.S.A.; 2 Comprehensive Epilepsy Center, Department of Neurology, NYP-Weill Cornell Medical Center, New York, NY U.S.A.; 3 Department of Neurology, Sir Ganga Ram Hospital, New Delhi, India; 4 Department of Medicine, Saint Vincent's Hospital and Medical Centers, New York, NY U.S.A.

Dear Editor,

Many new serotonergic antidepressants have been introduced into the market over the past decade. As compared to the older tricyclic antidepressants, these usually have a more favorable side-effect profile. As more experience is gained with these newer antidepressants new side-effects are being reported. Venlafaxine (Effexor) has been FDA-approved for the treatment of depression, social anxiety disorder, panic disorder and generalized anxiety disorder. Off-label uses include migraines, diabetic neuropathy and for peri-menopausal symptoms (hot flashes). Common side-effects reported with venlafaxine include nausea, dizziness, sleepiness, sweating, dry mouth, nervousness, insomnia, constipation, confusion, agitation and increased cholesterol. Less common but more serious side-effects such as tachycardia, confusion, changes in vision, mania and seizures have also been documented.[1] We present here a case of a woman who developed impaired detrusor contractility (IDC) due to venlafaxine use, which resolved on drug discontinuation.

A 48-year-old woman presented with complaint of difficulty in voiding urine of 11 days duration. She had a history of multilevel lumbar disk disease and reported lower back and leg pain that preceded the voiding issue. She was menopausal and experienced hot flashes after having recently discontinued hormone replacement therapy. Her only medication was venlafaxine 300 mg daily which she was talking for depression. Apart from tenderness in the lower back, the neurological examination was essentially normal. There was neither clinical evidence for neuropathy nor evidence of lumbosacral root dysfunction to explain her recent onset of difficulty in voiding urine. The magnetic resonance imaging scan was non-revealing. EMG and nerve conduction studies were normal while urodynamic studies were suggestive of IDC with post-void residual more than 200 ml. After considering the possibility of venlafaxine-induced IDC, she was tapered off the medication. Soon after the taper, patient reported return of normal bladder function. Follow-up urodynamic studies were declined by the patient.

Venlafaxine, a combined serotonin-norepinephrine reuptake inhibitor (SNRI), is a relatively new antidepressant drug. There have been reports of male patients experiencing urinary incontinence while taking venlafaxine.[2] Votolato *et al.*, described the case of a 51-year-old woman with bipolar I disorder on venlafaxine who developed urge incontinence requiring the use of four to six pads per day. To reduce her incontinence she found it necessary to void prophylactically every two hours. Her urge incontinence resolved when she was switched from venlafaxine to bupropion, a non-serotonergic antidepressant with noradrenergic and dopaminergic properties.[3] It is worthy to note that duloxetine (Cymbalta) which too is an SNRI is approved in the European Union for the treatment of stress urinary incontinence (SUI).[4] Duloxetine causes an increase in the urethral closure pressure. The authors reviewed all randomized controlled trials comparing duloxetine with placebo or no treatment. The duloxetine group had significantly better quality-of-life scores and rates of symptom improvement for participants on 80 mg daily (weighted mean difference for Incontinence Quality of Life Index for participants on 80 mg daily: 4.5; 95%CI, 2.83-6.18; *P*<0.00001). They concluded that duloxetine significantly improved the quality of life of patients with SUI, but it was unclear whether or not benefits are sustainable. While the mechanism of action by which venlafaxine causes IDC is unclear, *in vivo* experiments in animals have shown that both serotonin and norepinephrine have central nervous system effects that affect lower urinary tract function. We wish to add IDC to the list of possible urological side-effects associated with venlafaxine use.
